# Small Bowel Intussusception due to Metastasized Sarcomatoid Carcinoma of the Lung: A Rare Cause of Intestinal Obstruction in Adults

**DOI:** 10.1155/2012/962683

**Published:** 2012-12-30

**Authors:** Ali Guner, Savaş Karyagar, Ayten Livaoglu, Can Kece, Uzer Kucuktulu

**Affiliations:** ^1^Department of General Surgery, Trabzon Kanuni Training and Research Hospital, 61040 Trabzon, Turkey; ^2^Department of Nuclear Medicine, Trabzon Kanuni Training and Research Hospital, 61040 Trabzon, Turkey; ^3^Department of Pathology, Trabzon Kanuni Training and Research Hospital, 61040 Trabzon, Turkey

## Abstract

Although small bowel intussusception is one of the most common abdominal emergencies in childhood, it is rare in adults and usually occurs as a result of an underlying pathology. Sarcomatoid carcinoma, a very rare subtype of lung cancer, rarely metastasizes to small bowel and causes complications. In this paper, we aim to describe a patient with small bowel intussusception caused by an isolated small bowel metastasis of the sarcomatoid carcinoma of the lung by reviewing the literature.

## 1. Introduction

Bowel obstruction is a major cause of morbidity and financial expenditure in hospitals around the world and is frequently encountered in emergency departments. It can develop for a variety of etiological reasons, with adhesions being the most common cause. Intussusceptions constitute 1–5% of all cases of bowel obstructions. Despite intussusception being commonly observed in childhood, it is a rare cause of bowel obstruction in adult populations. Small bowel intussusception (SBI) in adults can be observed in association with idiopathic or benign causes, or they may develop secondary to malignant pathologies. In the latter case, the underlying causes of intussusception can be primary small intestine tumors, such as lymphoma, gastrointestinal stromal tumors and carcinoid tumors, or the metastasis of cancers to the small intestine from other organs [1].

## 2. Case Presentation

**Figure 1:**
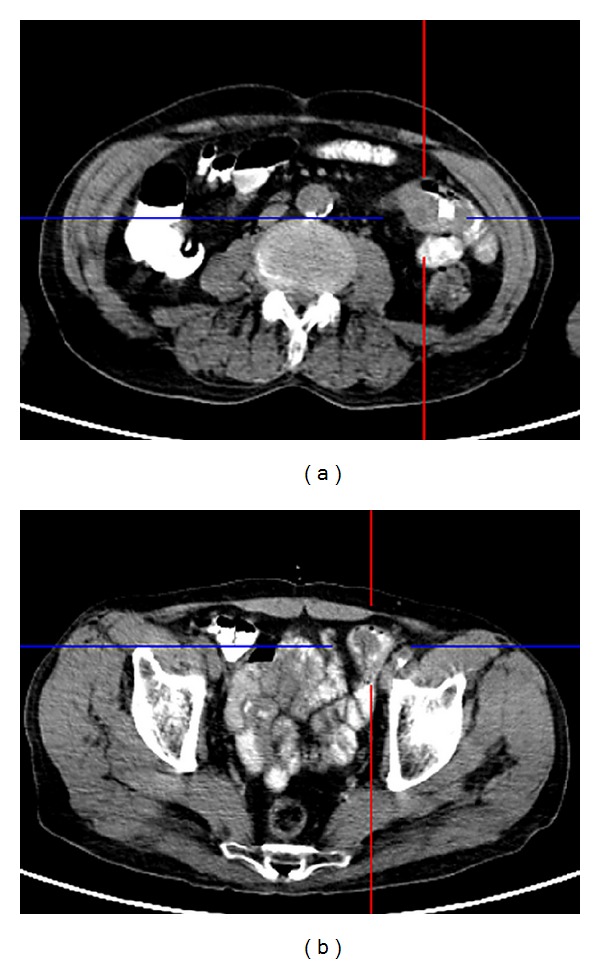
Abdominal CT image of tumors located in the small intestines.

**Figure 2:**
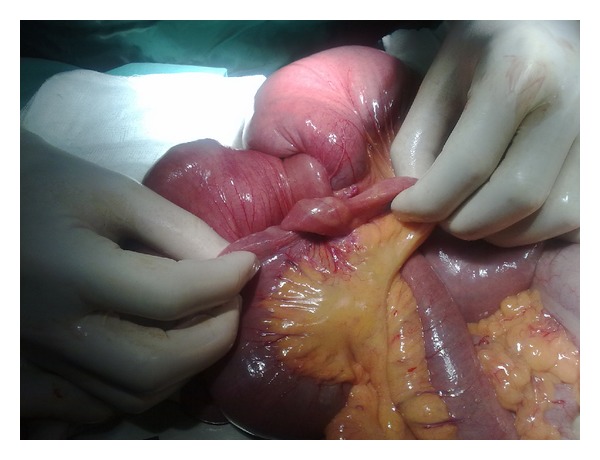
Image of intussuscepted small bowel caused obstructions in two different segments and dilated small bowel loops.

**Figure 3:**
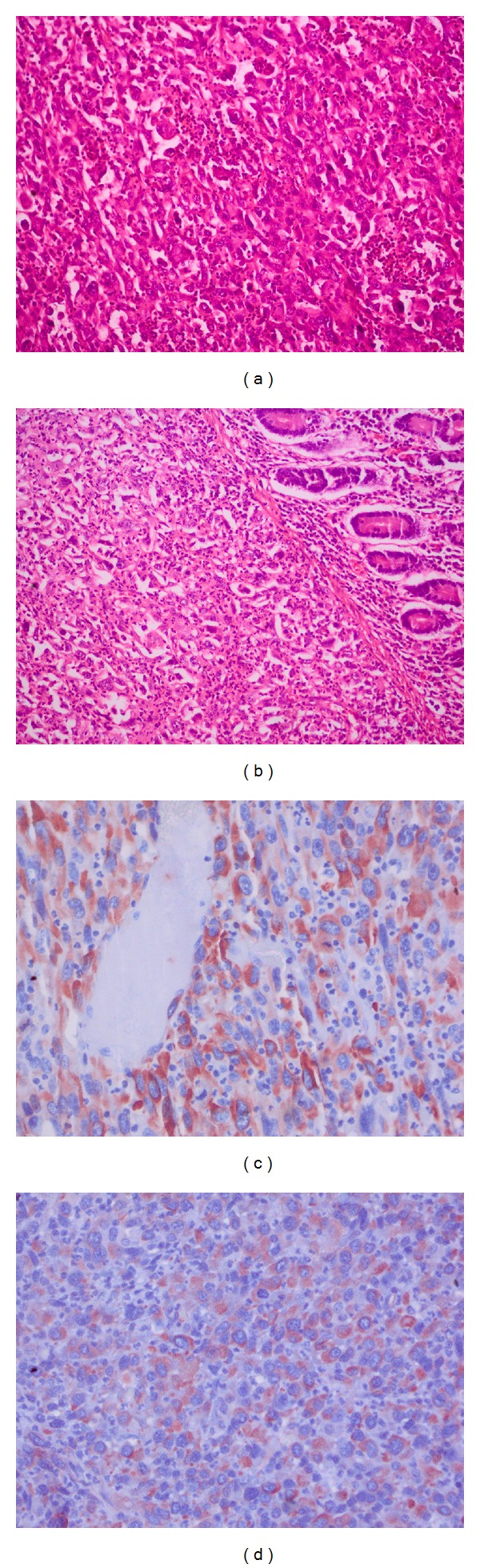
Pictures of histopathological and immunohistochemical results. (a) Epithelioid and fusiform shaped pleomorphic carcinoma infiltration in segments obtained from the lungs (hematoxylin-eosin, 20x magnification). (b) Submucosal neoplastic infiltration of similar morphology in segments obtained from the small intestines (hematoxylin-eosin, 20x magnification). (c) Strong cytoplasmic staining with cytokeratin in segments obtained from the small intestines (40x magnification). (d) Strong cytoplasmic staining with vimentin in segments obtained from the small intestines (40x magnification).

A 71-year-old man was admitted to our emergency department complaining of abdominal pain and vomiting. He described the absence of gas and feces for two days. Physical examination demonstrated a distended abdomen and mild epigastric tenderness. Auscultation was indicated by hyperactive bowel sounds and a plain abdominal film showed gas-fluid levels. Aside from a hemoglobin value of 6 and a hematocrit value of 21, laboratory evaluations revealed no pathological characteristics. Based on these observations, the patient was monitored with a diagnosis of anemia and mechanical intestinal obstruction. It was also learnt that the patient had undergone surgery one year previously for a lung tumor, and that he had been diagnosed with sarcomatoid carcinoma of the lung based on histopathological evaluations (pT3, pN1). The patient had had no further complaints in the following one year. Abdominal computerized tomography (CT) identified a mass located in the small intestines (Figure 1). Following preoperative preparations for the patient, a midline laparotomy was performed. Exploration revealed invagination in two different regions of the small bowel loops (Figure 2). Two separate segmental resections and primary anastomosis were performed. After opening the invaginated segments following resection, a mass with a polypoid appearance was observed. The patient had no postoperative complications or issues and was discharged with full recovery on the 7th day following surgery. During histopathological examination of the resected specimen, malignant tumor infiltration with localized fusiform cells and distinct pleomorphism was observed. Immunohistochemical evaluation revealed strong staining for vimentin, cytokeratin, and CK47, while no staining was observed for CD117, S-100 protein, HMB-45, Actin, Desmin, Synaptophysin, and CD34. As a result, small intestine metastasis of sarcomatoid carcinoma of the lung was reported (Figure 3). The patient died on the 9th postoperative month due to disease progression.

## 3. Discussion

Despite being the most common abdominal emergency in early childhood, intussusception is a rarely encountered pathology among adults. Starting from a mass or a lead point in the intestinal lumen, it is formed by telescopic invagination of the proximal loop into the distal loop. While intussusception in childhood is frequently idiopathic, in adults it commonly develops as a result of an underlying benign or malign etiology [2]. In the case series described by Chiang and Lin, adenocarcinoma was involved in the majority of colon-based cases, while cases of intussusceptions located in the small intestines generally revealed more benign etiologies [3]. In the presentation by Shi et al., 38 cases were reported as developing SBI due to metastatic disease between 1965 and 2007 [4]. Since 2007, a Medline search found 22 more cases that have been reported. The distribution of these cases is shown in Table 1. Despite 15 of these cases being reported as being associated with lung cancer metastasis, only one of these cases was related to sarcomatoid carcinoma of the lung. This presentation provides a discussion of the second case in the literature.

Lung cancer is the leading cause of cancer-related death worldwide. Lung carcinoma is capable of metastasis to other regions within the body. Patients are rarely symptomatic and can be diagnosed only following the development of complications such as obstruction, perforation, or hemorrhage. The proper evaluation of abdominal pathologies during follow-up of lung cancer is thus important. Despite Garwood et al. reporting on a series of 98 patients with lung cancer, with findings of gastrointestinal metastasis for adenocarcinoma (23.7%), squamous cell carcinoma (22.7%), large cell carcinoma (20.6%), and small cell carcinoma (19.6%), cases of intussusception associated with sarcomatoid carcinoma were very rare [5]. This pathological subtype has more aggressive clinical picture compared to other non-small cell cancers [6]. In a previously reported case (pT2, pN0), intussusception developed 17 months following the diagnosis of lung cancer, and the patient died on the 21st postoperative month. In the presented case, in which the disease was at a more advanced stage, symptoms were observed earlier and the survival period was correspondingly shorter.

Although it is known that lung carcinoma can metastasize to the small intestine via the hematogenous or lymphogenous paths, the exact route followed during metastasis is not known. However, retrograde metastasis to the lymphatic system of the small intestines via the thoracic duct appears possible. Immunohistochemical staining during histopathological evaluation is important for proper identification. In a review regarding sarcomatoid of the small bowel, strong positive results were observed with vimentin, cytokeratin AE1/AE3, and CK7, while negative staining was observed for the S100 protein, muscle cell actin, desmin, carsinoembryonic antigen, CK20, and CK117, which are important in differential diagnosis from other malignant pathologies [7]. Similar results were obtained in the immunohistochemical evaluation of the presented case.

The treatment plan for adult intussusception consists of segmental resection and primary restoration of continuity of the gastrointestinal tract. As malignant pathologies are frequently observed in cases of colonic intussusception, reduction is not recommended for these cases [8]. However, in cases of SBI it has been reported that resection can be performed following manual reduction to allow the resection of a smaller segment and to avoid the occurrence of short bowel syndrome. In SBI cases related to metastatic tumors there is always a risk of tumor perforation and complications that may arise due to ischemia in the intestinal mucosa. Therefore, resection without reduction will be more suitable in these cases. Due to the tendency of metastases to occur at multiple sites, the evaluation of all intestinal segments by palpation and, if possible, intraoperative enteroscopy can be considered.

In conclusion, it is important to consider that intestinal metastases of lung cancers can occur regardless of the type of pathology, that these metastases may have different clinical manifestations, and that the diagnostic and therapeutic approaches should be planned accordingly. Therefore, the existence of lung malignancies in the history of any patient presenting with abdominal symptoms should be evaluated carefully.

## Figures and Tables

**Table 1:** Reported cases of adult small bowel intussusception caused by metastatic disease.

	1965–2007 [4] (Number of patients)	After 2007 (Number of patients)	Total (Number of patients)
Malignant melanoma	16	8	24
Lung cancer	9	6	15
Renal cell cancer	4	2	6
Others	9	6	15
Esophageal cancer		1	
Gluteal leiomyosarcoma		1	
Pleural mesothelioma		1	
Blue rubber bleb naevus syndrome		1	
Peripheral nerve sheath tumor		1	
Liposarcoma of the thigh		1	
